# Elevation of brain magnesium prevents synaptic loss and reverses cognitive deficits in Alzheimer’s disease mouse model

**DOI:** 10.1186/s13041-014-0065-y

**Published:** 2014-09-13

**Authors:** Wei Li, Jia Yu, Yong Liu, Xiaojie Huang, Nashat Abumaria, Ying Zhu, Xian Huang, Wenxiang Xiong, Chi Ren, Xian-Guo Liu, Dehua Chui, Guosong Liu

**Affiliations:** School of Medicine, Tsinghua University, Beijing, China; Neuroscience Research Institute, Health Science Center, Peking University, Beijing, China; Department of Physiology, Zhongshan School of Medicine, Sun Yat-sen University, Guangzhou, China

**Keywords:** Alzheimer’s disease, Brain magnesium, Synaptoprotection, NMDAR signaling, BACE1

## Abstract

**Background:**

Profound synapse loss is one of the major pathological hallmarks associated with Alzheimer’s disease, which might underlie memory impairment. Our previous work demonstrates that magnesium ion is a critical factor in controlling synapse density/plasticity. Here, we tested whether elevation of brain magnesium, using a recently developed compound (magnesium-L-threonate, MgT), can ameliorate the AD-like pathologies and cognitive deficits in the APPswe/PS1dE9 mice, a transgenic mouse model of Alzheimer’s disease.

**Results:**

MgT treatment reduced Aβ-plaque, prevented synapse loss and memory decline in the transgenic mice. Strikingly, MgT treatment was effective even when the treatment was given to the mice at the end-stage of their Alzheimer’s disease-like pathological progression. To explore how elevation of brain magnesium ameliorates the AD-like pathologies in the brain of transgenic mice, we studied molecules critical for APP metabolism and signaling pathways implicated in synaptic plasticity/density. In the transgenic mice, the NMDAR signaling pathway was downregulated, while the BACE1 expression were upregulated. MgT treatment prevented the impairment of these signaling pathways, stabilized BACE1 expression and reduced sAPPβ and β-CTF in the transgenic mice. At the molecular level, elevation of extracellular magnesium prevented the high Aβ-induced reductions in synaptic NMDARs by preventing calcineurin overactivation in hippocampal slices.

**Conclusions:**

Our results suggest that elevation of brain magnesium exerts substantial synaptoprotective effects in a mouse model of Alzheimer’s disease, and hence it might have therapeutic potential for treating Alzheimer’s disease.

**Electronic supplementary material:**

The online version of this article (doi:10.1186/s13041-014-0065-y) contains supplementary material, which is available to authorized users.

## Background

Dysfunctions in the metabolic processes of amyloid precursor protein (APP) are widely hypothesized to underlie Alzheimer’s disease (AD) [1,2]. In this scenario, increases in concentration of several potentially toxic peptides including sAPPβ and β-CTF [3], N-APP [4] or small Aβ oligomers lead to the formation of Aβ-plaques, synapse dysfunction/loss, neuronal loss and overall brain atrophy, which cause decline of cognitive abilities [2,5]. Therefore, designing therapeutic agents that target APP metabolic processes is among the major strategies being pursued in the quest to treat AD [1]. However, these agents have not been shown to slow/reverse the cognitive deficits in AD patients [1].

Although dysfunctions in APP metabolism are a strong predictor for developing AD, Aβ-plaque density does not always correlate with the decline in cognitive abilities [6]. For example, some Aβ-plaque-positive individuals have no signs of brain atrophy [7], and highly educated subjects seem to tolerate high levels of Aβ-plaques without cognitive impairment [8]. Thus, while dysfunctions in APP metabolism might be necessary, it does not appear sufficient for developing AD-associated atrophy and cognitive impairment [9].

Numerous studies demonstrate that elevation of soluble Aβ in the brain could impair synaptic function and reduce synapse density [2,10]. However, synaptic plasticity and density are regulated by numerous endogenous factors [11], with some having neuro-/synapto-trophic functions. Hence, a balance between APP-derived neurotoxic peptides and neuro-/synapto-trophic factors might ultimately determine the rate of decline in synaptic and cognitive functions in AD. Indeed, several endogenous neurotrophic factors are effective in counteracting the synapse and memory loss in animal models of AD. For example, administration of the peptide cerebrolysin [12], the octapeptide NAP [13], nerve growth factor (NGF) [14], or brain infusion of brain-derived neurotrophic factor (BDNF) [15], in rodent and primate AD models reverses synapse loss or rescues learning and memory. In humans, intranasal administration of insulin improves cognition in early AD [16]. Therefore, protecting existing synapses and/or promoting synapse generation/regeneration by increasing trophic factors in brain might present an alternative strategy to ameliorate memory deficits in AD.

Our previous studies indicate that the magnesium ion (Mg^2+^) is a critical factor in controlling synapse density/plasticity [17]. We have shown that elevation of brain magnesium by a novel compound (Mg-L-threonate, MgT) upregulates NMDAR signaling, prevents synapse loss and reverses memory deficits in aged rats [18]. If impairment in cognitive functions in AD patients is mainly caused by synapse failure/loss [19], it becomes tempting to test whether elevation of brain magnesium could still be effective under AD-like pathological conditions. Furthermore, brain magnesium level [20] and serum Mg^2+^ concentration [21] appear to be significantly lower in AD patients compared with age-matched normal subjects. If so, then simply restoring brain magnesium might beneficiate AD patients. Here, we show that elevation of brain magnesium prevents/reverses synapse loss and memory deficits in a transgenic mouse model of AD and reveal the possible underlying mechanism.

## Results

### Elevation of brain magnesium prevented learning and memory deficits in APPswe/PS1dE9 transgenic mice

**Figure 1:**
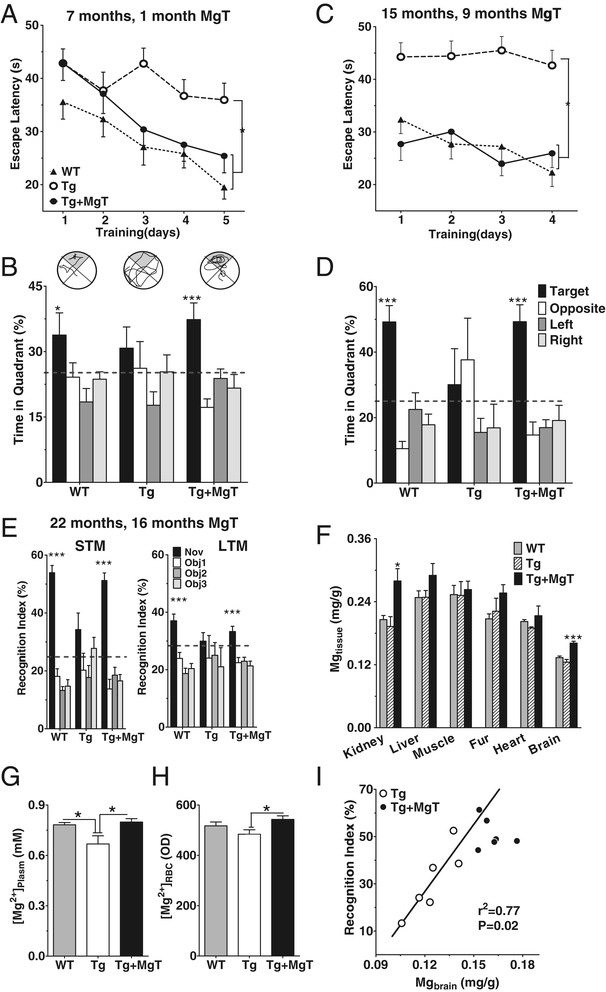
**Prevention of memory deficits in APPswe/PS1dE9 transgenic mice (Tg mice) by MgT treatment. (A)** Escape latencies in seconds(s) during training (5 trials/day) in water maze task at 7 months of age, i.e., after 1 month of treatment. Three groups of mice were used; WT (*n* = 9), Tg (*n* = 13) and Tg + MgT (*n* = 9; ANOVA effect of treatment, *p* < 0.05). **(B)** Probe test conducted 24 h after the training. Top, representative path tracings. Bottom, percentage of time spent in each quadrant (ANOVA differences among quadrants; WT: *p* < 0.05; Tg + MgT: *p* < 0.0001). **(C)** Same as **(A)** and on the same mice, but tested at 15 months of age. WT (*n* = 9); Tg (*n* = 7); Tg + MgT (*n* = 6). ANOVA effect of treatment *p* < 0.05. **(D)** Probe test 24 h later (ANOVA differences among quadrants: WT: *p* < 0.0001; Tg + MgT: *p* < 0.0001). **(E)** Short-term (10 m in retention interval, left) and long-term (24 h, right) novel-object recognition memory tests performed on the same group of mice at 22 months of age. WT (*n* = 7), Tg (*n* = 6), and Tg + MgT (*n* = 6). Recognition index calculated as percentage of time spent exploring each object (Obj1-3). Black bars indicate novel object (Nov). ANOVA differences in recognition index of different objects in WT (STM: *p* < 0.0001; LTM: *p* < 0.0001) and Tg + MgT (STM: *p* < 0.0001; LTM: *p* < 0.001). Dashed lines represent chance levels of performance (25%). **(F)** Total magnesium (ionized and non-ionized) contents in different organs/tissues (Mg_tissue_) normalized to tissue weight (mg/g) in the same groups of mice. WT (*n* = 7), Tg (*n* = 6) and Tg + MgT (*n* = 6). ANOVA difference among groups (brain: *p* < 0.0001; kidney: *p* < 0.01). **(G)** Magnesium ion concentration in the plasma ([Mg^2+^]_plasma_, mM) of WT (n = 14), Tg (n = 9) and Tg + MgT mice (n = 8) as measured by the calmagite method. ANOVA difference among groups (*p* < 0.01). **(H)** The intracellular free Mg^2+^ concentration in the red blood cell ([Mg^2+^]_RBC_) of WT (n = 12), Tg (n = 11) and Tg + MgT mice (n = 11) as measured by the flow cytometry method (fluorescent optical density, OD). ANOVA difference among groups (*p* < 0.05). ANOVA was followed by Bonfferoni’s *post hoc* test. **(I)** Brain total magnesium content (Mg_brain_, mg/g), in Tg mice (23 months old), significantly correlated with the recognition index in the short-term recognition memory test (*Pearson’s* test). Data from Tg + MgT mice (23 months old treated for 17 months) are displayed, but were not included in the correlation analysis. Error bars show SEM. * *p* < 0.05, *** *p* < 0.001.

We chose the APPswe/PS1dE9 transgenic mouse as an animal model of AD (referred to as Tg mice) that exhibits high brain amyloid deposits [22] and severe deficits in spatial memory at 6–7 months of age [23–25]. In our previous study in rats, the effective dosage for memory enhancement by MgT was 50 mg/kg/day elemental Mg [18]. The equivalent elemental magnesium dose in mice is 75 mg/kg/day (910 mg/kg/day MgT) [26]. This dose was effective in elevating Mg^2+^ concentration in plasma and red blood cells (Figure 1G and H). Interestingly, we found that Mg^2+^ concentration in the plasma of untreated Tg mice was significantly lower than that of WT mice (Figure 1G), those data are inline with previous studies showing reductions in Mg^2+^ concentration in the serum of AD patients [21].

We tested whether elevating brain magnesium could prevent learning and memory deficits in Tg mice. MgT treatment started at 6 months of age. After 1 month of MgT treatment, we assessed spatial learning and memory abilities using the water maze task. Untreated Tg mice exhibited unequivocal learning deficits in this task at 7 months of age. MgT-treated Tg mice (Tg + MgT), in contrast, performed quite similar to WT (Figure 1A). When we did a probe test 24 h after the last training trial, untreated Tg mice showed no preference toward the target quadrant, indicating significant memory impairment, while Tg + MgT mice performed as well as WT (Figure 1B).

The improvement of cognitive functions after 1 month treatment with MgT is interesting. However, from therapeutic point of view, efficacy of MgT on cognitive function over a longer time-course is more important. To check if MgT remained effective over a long-term treatment, the same groups of mice were retested at 15 and 22 months of age (after 9 and 16 months of MgT treatment, respectively). At 15 months, learning and memory abilities were reassessed using the same water maze task (modified protocol, see Methods). Tg mice completely lost their ability to locate the hidden platform, while Tg + MgT and WT mice could readily locate it (Figure 1C). In the probe test, Tg + MgT mice, similar to WT, spent significantly more time in the target quadrant compared with other quadrants, while untreated Tg mice swam randomly (Figure 1D). We did not observe significant differences in the velocity, in the water maze, among the three groups of mice at both ages of 7 and 15 months (Table 1), indicating that the differences in latencies/time-in-quadrant among the groups were not because the untreated Tg mice swam slower than other mice.

**Table 1:** **Body weight, food/fluid intake, and locomotor activity in the open field of wild-type (WT, n = 7), Tg (n = 6) and Tg + MgT (n = 6) mice and the velocity in the water maze task of WT, Tg and Tg + MgT mice at age of 7 months (n = 9, 13, 9; respectively) and 15 months (n = 9, 7, 6; respectively)**

**Group of mice**	**Body weight (g)**	**Food intake (g/day)**	**Fluid intake(ml/day)**	**Locomotor activity in the open field**		**Velocity in the water maze (cm/s)**	
				**Velocity (cm/s)**	**Distance travelled (cm)**	**7 months old**	**15 months old**
WT	42.84 ± 3.92	4.08 ± 0.61	7.16 ± 0.26	5.72 ± 0.75	3403.99 ± 443.13	11.2 ± 1.1	12.9 ± 2.6
Tg	40.87 ± 10.80	3.41 ± 1.48	6.83 ± 0.71	4.34 ± 0.48	2573.52 ± 286.99	9.9 ± 1.1	11.1 ± 1.9
Tg + MgT	39.73 ± 3.57	3.69 ± 0.74	6.25 ± 1.00	5.03 ± 0.32	2954.00 ± 159.68	10.8 ± 1.5	11.8 ± 2.4

On way ANOVA revealed no significant differences among the three groups of mice. Data presented as mean ± SD.

At 22 months, we assessed short- and long-term recognition memory (STM and LTM) using a modified version of the standard novel-object recognition task (NORT). The NORT was selected to avoid repeating the water maze task for the third time and because the mice were too weak to perform the water maze task at age of 22 months. During the STM test (10 m retention interval), Tg mice did not show any preference toward the novel object. In contrast, WT and Tg + MgT mice spent significantly more time exploring the novel object (Figure 1E, left). During the LTM test (24 h retention interval) only WT and Tg + MgT mice exhibited clear preference toward the novel object (Figure 1E, right). Thus, MgT treatment remained effective after long-term (up to 16 months) treatment.

To confirm that MgT treatment was associated with elevating brain magnesium, we quantified the magnesium contents (total magnesium in tissue) in brain and in other organs in the behaviorally tested mice mentioned above (perfused at age of 23 month). MgT treatment selectively elevated Mg^2+^ content in brain and kidneys (Figure 1F) of Tg mice. Interestingly, in untreated Tg mice, brain magnesium level positively correlated with their cognitive function; the lower their brain magnesium, the poorer their memory function in the NORT task (Pearson’s test, *r*^2^ = 0.77, *p* = 0.02, Figure 1I). We did not observe significant side effects over the entire time-course of MgT treatment (Table 1).

### Elevation of brain magnesium prevented synapse loss in APPswe/PS1dE9 transgenic mice

**Figure 2:**
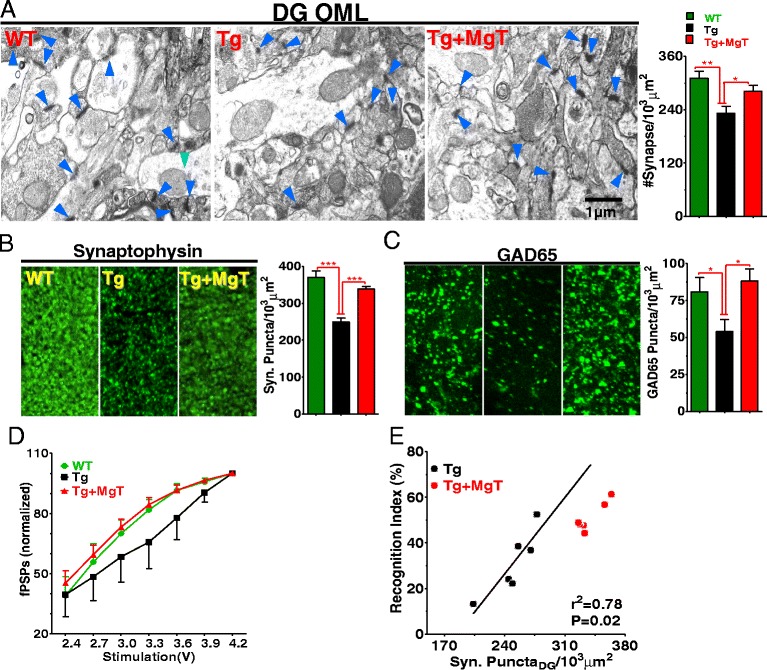
**Prevention by MgT treatment of synapse loss in APPswe/PS1dE9 transgenic mice (Tg mice). (A)** Left: electron microscopic images showing structural synapses (blue arrows) in hippocampal outer molecular layer of dentate gyrus (DG-OML). Right: Estimated synaptic density. WT (*n* = 6), Tg (*n* = 6) and Tg + MgT (*n* = 5). **(B)** Left: Immunostaining of synaptophysin-positive terminals (Syn Puncta) in DG-OML. Right: Quantitative analysis of Syn Puncta (*n* = 6/group). **(C)** Same as in **(B)** and from same groups of mice; however, puncta represent GABAergic (GAD65). **(D)** Input–output (normalized) relationship of hippocampal CA1 synapses in vivo (*n* = 6/group). Field post synaptic potentials (fPSPs) were normalized by the maximum amplitude of fPSPs. Two Way ANOVA revealed significant effects of treatment: p < 0.05; and stimulus: p < 0.0001. **(E)** Correlation between the density of Syn Puncta and short-term recognition memory in Tg mice (23 months old). Tg + MgT (23 months old treated for 17 months) data are displayed but were not included in the regression analysis (*Pearson’s* test). Error bars show SEM. * *p* < 0.05, ** *p* < 0.01, *** *p* < 0.001.

To study the cellular mechanisms underlying the prevention of memory deficits in Tg mice by MgT, we examined effects of this treatment on synapse density. The brains of the same mice used in the above-described behavioral experiments (Figure 1) were subjected to histological analysis. First, we quantified synapse density in the hippocampus, a brain region critical for memory function. Tg mice had a significantly lower number of synapses (by ~25.3%) compared to WT; this synapse loss was prevented by MgT treatment (Figure 2A, for ANOVA analysis see Table 2). We also checked the total presynaptic terminal density, using the synaptic vesicle marker synaptophysin, in the hippocampal dentate gyrus (DG). In Tg mice there were significantly fewer synaptic puncta (~32.5%) than those in WT mice. MgT treatment prevented this reduction in Tg mice (Figure 2B and Table 2).

**Table 2:** **One-way ANOVA analysis of the electron microscopy, immunostaining and Western blot data**

**Value**	**Basal**			**Stimulation**		
	**F value**	**P value**	**Figure**	**F value**	**P value**	**Figure**
**Protein**						
Structural synapses	**F**_**(2,14)**_ **=** 7.243	0.0069	Figure 2A	NA	NA	NA
Synaptophysin	**F**_**(2,15)**_ **=** 26.21	<0.0001	Figure 2B	NA	NA	NA
GAD65	**F**_**(2,15)**_ **=** 4.130	0.0373	Figure 2D	NA	NA	NA
NR2B/GAPDH	**F**_**(2,12)**_ **=** 1.026	0.3877	Figure 3B	**F**_**(2,14)**_ **=** 55.26	<0.0001	Figure 3B
pCamkII/CamkII	**F**_**(2,12)**_ **=** 0	1	Figure 3C	**F**_**(2,14)**_ **=** 4.269	0.0357	Figure 3C
pCreb/Creb	**F**_**(2,12)**_ **=** 0	1	Figure 3D	**F**_**(2,14)**_ **=** 9.883	0.0021	Figure 3D
BACE1/GAPDH	**F**_**(2,12)**_ **=** 28.70	< 0.0001	Figure 5C	**F**_**(2,14)**_ **=** 60.76	<0.0001	Figure 5C
Synaptophysin	NA	NA	NA	**F**_**(2,16)**_ **=** 20.15	<0.0001	Figure 7F

NA: not applicable.

Next, we examined the effects of MgT treatment on GABAergic terminals by quantifying density of glutamic acid decarboxylase 65 kD (GAD65) labeled puncta. Tg mice had significantly lower immunostained puncta density for GAD65 (~33.3%, Figure 2C and Table 2) than WT. MgT treatment prevented the loss in GABAergic terminals (Figure 2C).

To determine the functional consequences of synapse density reduction on glutamatergic synaptic transmission, we compared the input–output relationship of hippocampal CA1 synapses in vivo, in a separate group of mice (15 months old, MgT treatment for 9 months). The amplitude of field postsynaptic potentials (fPSPs) for a given stimulus intensity was significantly lower in Tg mice compared to WT, while Tg + MgT mice had a similar input–output relationship as WT (Figure 2D). Therefore, MgT treatment was also effective in preventing reductions in glutamatergic synaptic transmission.

Finally, we plotted the relationship between synapse density and short-term memory (quantified by the recognition index during NORT task) in Tg mice. Memory performance correlated with the density of synaptophysin puncta (*r*^2^ = 0.78, *p* = 0.02, Figure 2E), suggesting that synapse loss might be one of primary factors underlying memory deficits in Tg mice.

### Impairment of NMDAR signaling in APPswe/PS1dE9 transgenic mice

During learning and memory processes, correlated synaptic inputs activate NMDARs, leading to activation of downstream signaling molecules and increase in expression of genes necessary for synaptic remodeling [27]. Having demonstrated that Tg mice have significant reductions in structural synapse density and glutamatergic synaptic transmission, we next evaluated whether activation of NMDAR signaling still occurs in the remaining synapses following environmental enrichment behavioral stimulation. The environmental enrichment-based stimulus was chosen because it will evoke physiologic neuronal inputs.

**Figure 3:**
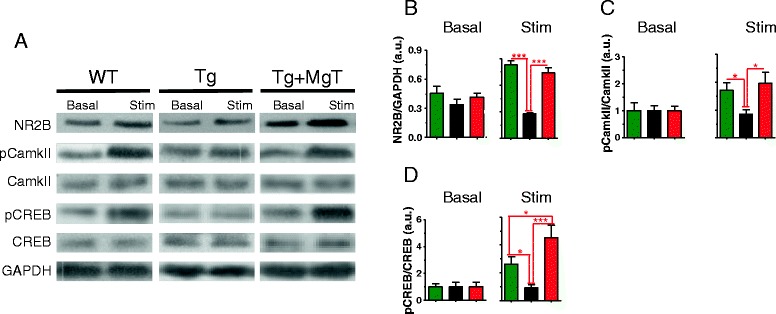
**Prevention by MgT treatment of impairment in NMDAR signaling pathway in APPswe/PS1dE9 transgenic mice (Tg mice). (A)** Representative Western blots showing the expression levels of NR2B, pCaMKII, CaMKII, pCREB, and CREB in the hippocampus of mice sacrificed either after home-cage conditions (basal) or following 24 h of environmental enrichment-based behavioral stimulation (Stim). GAPDH expression served as loading control. **(B)** Left: Quantitative analysis of NR2B expression in the hippocampus of WT, Tg and Tg + MgT mice (*n* = 5/group), obtained by Western blot (arbitrarily units, a.u.). Mice were sacrificed under basal conditions without behavioral stimulation. Right: Quantitative analysis of NR2B expression the hippocampus of WT (*n* = 6), Tg (*n* = 6) and Tg + MgT mice (*n* = 5) obtained by Western blot following behavioral stimulation. **(C and D)** same as (B) but quantifying Phosphorylated CaMKII (pCaMKII)/CaMKII ratio **(C)** and Phosphorylated CREB (pCREB)/CREB ratio **(D)**. ANOVAs (Table 2) were followed by Bonferroni’s *post hoc* test. Error bars show SEM. * *p* < 0.05, *** *p* < 0.001.

We prepared new groups of mice (sacrificed at age of 18 month, MgT treatment was given for 12 months) and compared the expression/phosphorylation of NMDAR signaling key proteins using quantitative Western blotting. Mice from each group were divided into two halves, one half was sacrificed under home-cage conditions (basal) and the second half was sacrificed following 24 h of environmental enrichment-based stimulation. Under basal conditions, the NMDAR signaling appeared to be identical between WT and Tg mice. Interestingly, environmental stimulation induced increases in NR2B expression (by 167%, Figure 3A and B), CaMKII activation (pCaMKII, by 129%, Figure 3A and C) and in CREB activation (pCREB, by 399%, Figure 3A and D) in WT but not in Tg mice. MgT treatment rescued these deficits (Figure 3 and Table 2). Thus, dysfunction of APP metabolism not only triggered synapse loss and reduction of synaptic transmission, but also impaired activation of NMDAR signaling by correlated synaptic inputs, which might result in loss of the plasticity in the remaining synapses. The expression/activation pattern of NMDAR signaling in Tg + MgT mice was almost identical to WT, in both basal and stimulated conditions. Therefore, MgT treatment might protect NMDAR signaling necessary for synaptic plasticity following neuronal activity.

### Prevention of exogenous Aβ42-induced downregulation of NMDAR synaptic transmission by elevation of [Mg^2+^]_o_

It is clear that MgT treatment not only prevented synapse loss in Tg mice, but also restored the functionality of NMDAR signaling in the remaining synapses. Since NMDARs are essential for synaptic plasticity and memory functions, we conducted the following experiments to explore, mechanistically, how elevation of Mg^2+^ can protect NMDARs from Aβ-induced downregulation.

**Figure 4:**
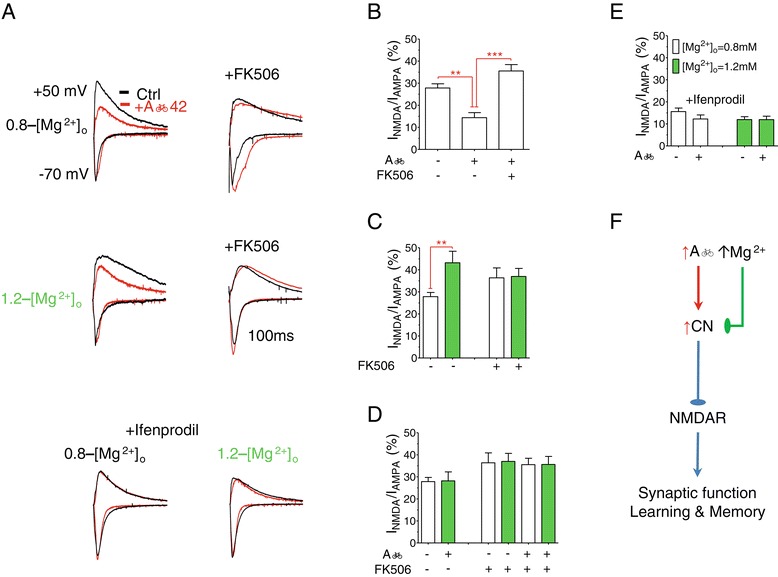
**Prevention of exogenous Aβ** **42-induced downregulation of synaptic NMDARs by elevation of [Mg** ^**2+**^ **]**_**o**_ **. (A)** Top panel: Representative traces of EPSCs recorded at membrane potentials of −70 and +50 mV at 0.8 mM [Mg^2+^]_o_ (0.8-[Mg^2+^]_o_) before (black) and after (red) application of Aβ42 with (right) or without (left) the addition of calcineurin inhibitor (FK506). Middle panel: Same as above but at 1.2 mM [Mg^2+^]_o_ (1.2-[Mg^2+^]_o_). Lower panel: EPSCs at 0.8 and 1.2 mM [Mg^2+^]_o_ with and without Aβ42 monomers in the presence of NR2B blocker (ifenprodil). **(B)** The ratio of amplitudes of EPSC_NMDAR_ to EPSC_AMPA_ (I_NMDA/AMPA_) in 0.8-[Mg^2+^]_o_ (*n* = 8), 0.8-[Mg^2+^]_o_ + Aβ42 (*n* = 7) and 0.8-[Mg^2+^]_o_ + Aβ42 + FK506 (*n* = 8) slices. ANOVA revealed significant difference among the groups (*p* < 0.0001). ANOVA was followed by Bonferroni’s *post hoc* test. ** *p* < 0.01, ***p < 0.001. **(C)** The ratio of amplitudes of EPSC_NMDAR_ to EPSC_AMPA_ (I_NMDA/AMPA_) in 0.8-[Mg^2+^]_o_ (*n* = 8), 1.2-[Mg^2+^]_o_ (green, *n* = 6), 0.8-[Mg^2+^]_o_ + FK506 (*n* = 7) and 1.2-[Mg^2+^]_o_ + FK506 slices (*n* = 7). **(D)** The ratio of amplitudes of EPSC_NMDAR_ to EPSC_AMPA_ (I_NMDA/AMPA_) in 0.8-[Mg^2+^]_o_ (*n* = 8), 1.2-[Mg^2+^]_o_ + Aβ42 (*n* = 6), 0.8-[Mg^2+^]_o_ + FK506 (*n* = 7) and 1.2-[Mg^2+^]_o_ + FK506 slices (*n* = 7), 0.8-[Mg^2+^]_o_ + Aβ42 + FK506 (*n* = 8) and 1.2-[Mg^2+^]_o_ + Aβ42 + FK506 (*n* = 8). **(E)** The ratio of amplitudes of EPSC_NMDAR_ to EPSC_AMPA_ (I_NMDA/AMPA_) after the addition of ifenprodil; 0.8-[Mg^2+^]_o_ (*n* = 6), 0.8-[Mg^2+^]_o_ + Aβ42 (*n* = 10), 1.2-[Mg^2+^]_o_ (*n* = 8) and 1.2-[Mg^2+^]_o_ + Aβ42 (*n* = 12). Recordings were conducted, in vitro, using acute hippocampal slices from 4 weeks old WT mice. Unpaired *t*-tests, ** *p* < 0.01. Error bars show SEM. **(F)** Schematic illustration of how high Aβ impairs NMDARs and how elevation of [Mg^2+^]_o_ might prevent this impairment.

First, we studied the molecular mechanisms underlying impairment of NMDARs by Aβ. The EPSC_NMDA_/EPSC_AMPA_ between CA3-CA1 synaptic connections (Shaffer collaterals) was recorded in hippocampal slices using whole-cell patch-clamp recordings from CA1 pyramidal neurons while stimulating Shaffer collaterals at low frequency (0.03 Hz). Exogenous application of Aβ (1 μM for 1 h at room temperature) significantly reduced I_NMDA_ (by ~48%), when brain slices were bathed under physiological extracellular magnesium concentration ([Mg^2+^]_o_ = 0.8 mM, referred to as 0.8-[Mg^2+^]_o_ slices, Figure 4B). A previous study suggests that downregulation of NMDARs by exogenous Aβ is likely mediated by overactivation of calcineurin [28]. We confirmed their observations and found that inhibition of calcineurin (FK506, 10 μM for 1 h) was effective in preventing downregulation of NMDARs by Aβ in hippocampal slices (Figure 4B). Therefore, calcineurin might be the molecular target mediating the Aβ-induced downregulation of NMDARs.

Next, we tested whether calcineurin is also involved in the molecular mechanism underlying the upregulation of NMDARs by elevation of [Mg^2+^]_o_ [17,18]. As expected, elevation of [Mg^2+^]_o_ (to 1.2 mM, 1.2-[Mg^2+^]_o_ slices), increased I_NMDA_ (by ~53%, percentage of 0.8-[Mg^2+^]_o_ slices, Figure 4C). Interestingly, in the presence of the calcineurin inhibitor, I_NMDA_ increased in 0.8-[Mg^2+^]_o_ (Figure 4C), while I_NMDA_ in 1.2-[Mg^2+^]_o_ remained the same such that the amplitudes of I_NMDA_ under 0.8-[Mg^2+^]_o_ and 1.2-[Mg^2+^]_o_ slices were almost identical (Figure 4C). Those data imply that elevation of [Mg^2+^]_o_ might upregulate I_NMDA_ by inhibiting calcineurin signaling. If both Aβ and [Mg^2+^]_o_ influence I_NMDA_ by controlling the activity of calcineurin, then elevation of brain magnesium might be able to prevent overactivation of calcineurin by high Aβ, resulting in protection of NMDARs. Indeed, in the presence of higher [Mg^2+^]_o_ (1.2 mM), the amplitude of I_NMDA_ current under exogenous Aβ was almost identical to that in 0.8-[Mg^2+^]_o_ control slices (incubated under Aβ free and physiological [Mg^2+^]_o_ conditions, Figure 4D). To prove that this protection is mediated by calcineurin, we compared the size of I_NMDA_ under high Aβ, high/low [Mg^2+^]_o_, or both in the presence of calcineurin inhibitor. I_NMDA_ was identical in all slices regardless of the presence of high Aβ or [Mg^2+^]_o_ (Figure 4D). Thus, elevation of brain magnesium might protect NMDARs in the Tg mice by preventing over-activation/-expression of calcineurin (Figure 4F).

Finally, we studied the effects of Aβ on I_NMDA_ after blocking NR2B-containing NMDARs with ifenprodil (3 μM for 10 m before recording). NR2A-containing NMDARs in 0.8- and 1.2-[Mg^2+^]_o_ slices were unaffected by Aβ (Figure 4E), suggesting that the reduction of I_NMDA_ after Aβ application was largely due to reduction of synaptic NR2B-portion of NMDARs, whereas elevated -[Mg^2+^]_o_ prevented this downregulation.

### Effects of elevating brain magnesium on amyloid plaques and BACE1 expression

**Figure 5:**
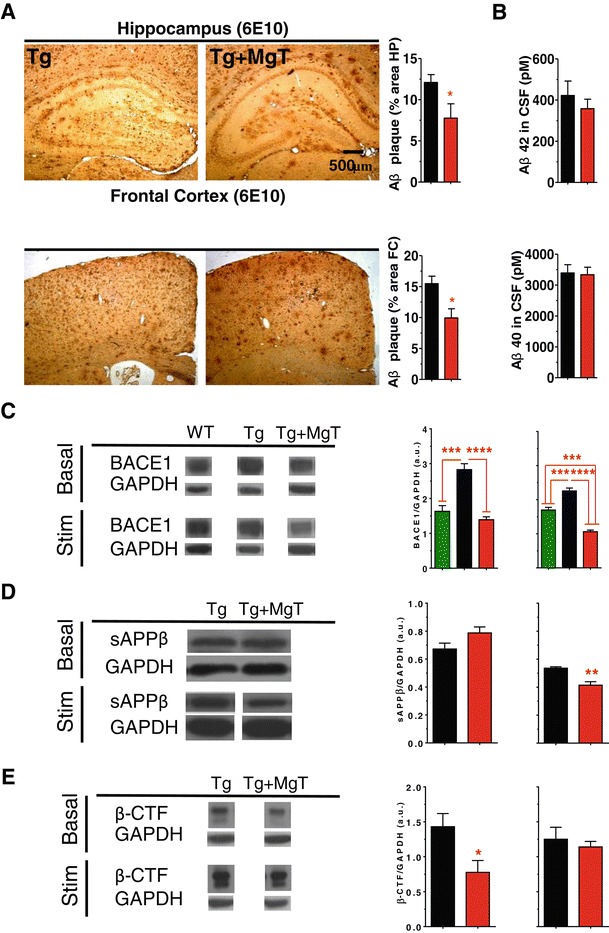
**Reductions by MgT treatment in amyloid plaques and BACE1 overexpression in APPswe/PS1dE9 transgenic mice (Tg mice). (A)** Upper panel left: Immunostaining of hippocampal amyloid plaque of Tg (*n* = 6) and Tg + MgT (*n* = 5). Right: Hippocampal amyloid plaque areas were significantly lower in Tg + MgT mice. Lower panel: same as above but in the frontal cortex. Two tailed unpaired *t*-test, * *p* < 0.05. **(B)** Concentrations of Aβ42 (upper panel) and Aβ40 (lower panel) monomers in CSF of Tg (*n* = 13) and Tg + MgT (*n* = 15) measured by ELISA. **(C)** Left: Representative Western blots of BACE1 (β-secretase) expression in the hippocampus of mice sacrificed under home-cage conditions (basal) or following 24 h of environmental enrichment-based stimulation (Stim). Middle: Quantitative analysis of BACE1 expression in the hippocampus of WT, Tg and Tg + MgT mice (*n* = 5/group) obtained by Western blot (arbitrarily units, a.u.). Mice were sacrificed under basal conditions without behavioral stimulation. Right: Quantitative analysis of BACE1 expression in the hippocampus of WT (*n* = 6), Tg (*n* = 6) and Tg + MgT mice (*n* = 5) obtained by Western blots following behavioral stimulation. ANOVAs (Table 2) were followed by Bonferroni’s *post hoc* test. **(D)** Left: Representative Western blots of hsAPPβ expression in the hippocampus of mice sacrificed under home-cage conditions (basal) or following 24 h of environmental enrichment-based stimulation (Stim). Middle: Quantitative analysis of hsAPPβ expression in the hippocampus of Tg and Tg + MgT mice (*n* = 5/group) obtained by Western blot (arbitrarily units, a.u.). Mice were sacrificed under basal conditions without behavioral stimulation. Right: Quantitative analysis of hsAPPβ expression in the hippocampus of Tg (*n* = 6) and Tg + MgT mice (*n* = 5) obtained by Western blots following behavioral stimulation. **(E)** Same as **(D)** but for β-CTF, Tg (n = 3) obtained by Western blots following behavioral stimulation. Two tailed unpaired *t*-tests. Data from WT mice was displayed but not included in the analysis. Error bars show SEM. * *p* < 0.05, ** *p* < 0.01, *** *p* < 0.001.

Having demonstrated that elevating brain magnesium can protect NMDAR signaling, we explored whether elevation of brain magnesium can affect APP metabolism, which might also contribute to the retained cognitive abilities in MgT-treated Tg mice. We, first, quantified amyloid plaques density, using the anti-Aβ-antibody 6E10, in the hippocampus and frontal cortex. At 23 months of age, ~12% of the hippocampal area in Tg mice was occupied by amyloid plaques. MgT treatment (for 17 months) reduced the amyloid plaque area significantly (by ~35.8%, Figure 5A, upper panel). A similar percentage of reduction was observed in the frontal cortex (by ~36%, Figure 5A, lower panel). These observations were confirmed by another anti-Aβ-antibody (4G8, data not shown). We checked the concentrations of Aβ42 and Aβ40 monomers in the cerebrospinal fluid (CSF) of a separate group of Tg mice (14 months old, MgT treatment for 8 months). The concentration of Aβ42 was elevated in the CSF of Tg mice. Surprisingly, despite the significant reduction in the amyloid plaques density, CSF concentration of both Aβ monomers was unaltered by MgT treatment (Figure 5B).

To study the molecular mechanisms underlying the reduction of amyloid plaques by MgT treatment, we investigated the expression of β-secretase (BACE1) in the three groups of mice using quantitative Western blot analysis (brain homogenates from same mice in Figure 3, see the Additional file 1: Figure S1 and Additional file 2: Figure S2 for the raw data). Under home-cage and following environmental enrichment-based stimulation, Tg mice had significantly higher BACE1 expression levels than WT mice (Figure 5C). Interestingly, MgT treatment prevented overexpression of BACE1 under both basal and stimulated conditions in Tg mice (Figure 5C and Table 2). To check if the reductions in BACE1 by MgT lead to reductions in the synaptotoxic products of BACE1; namely sAPPβ and β-CTF [3], we investigated the expression of human sAPPβ and β-CTF in Tg and Tg + MgT mice. We found that Tg + MgT mice had significantly lower sAPPβ expression levels than untreated Tg mice following environmental enrichment-based stimulation (Figure 5D). Quantitative Western blot analysis also showed that Tg + MgT mice had significantly lower β-CTF levels than untreated Tg mice under basal conditions (Figure 5E). Thus, MgT treatment might reduce the production of APP synaptotoxic metabolites by stabilizing the expression of BACE1 in Tg mice. These results uncover a new and intriguing potential molecular target of elevated brain magnesium in APP metabolism.

**Figure 6:**
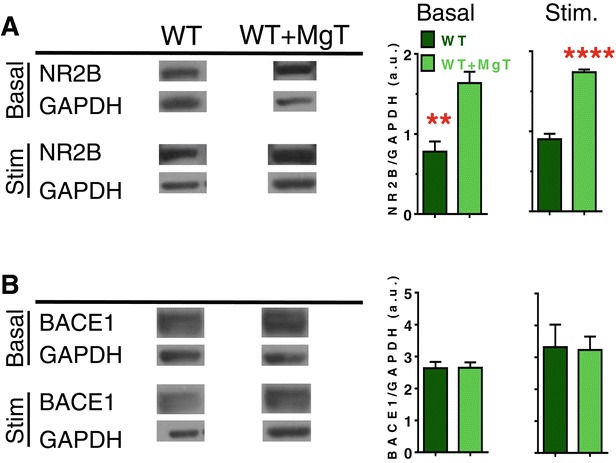
**Effects of MgT treatment on expression of NR2B and BACE1 in wild type mice. (A)** Left: Representative Western blots of NR2B expression in the hippocampus of WT mice sacrificed under home-cage conditions (basal) or following 24 h of environmental enrichment-based stimulation (Stim.). Middle: Quantitative analysis of NR2B expression in the hippocampus of WT and WT + MgT mice (*n* = 5/group) obtained by Western blot (arbitrarily units, a.u.). Mice were sacrificed under basal conditions without behavioral stimulation. Right: Quantitative analysis of NR2B expression in the hippocampus of WT and WT + MgT mice (*n* = 6/group) obtained by Western blots following behavioral stimulation. **(B)** Left: Representative Western blots of BACE1 (β-secretase) expression in the hippocampus of WT mice sacrificed under home-cage conditions (basal) or following 24 h of environmental enrichment-based stimulation (Stim.). Middle: Quantitative analysis of BACE1 expression in the hippocampus of WT and WT + MgT mice (*n* = 5/group) obtained by Western blot (arbitrarily units, a.u.). Mice were sacrificed under basal conditions without behavioral stimulation. Right: Quantitative analysis of BACE1 expression in the hippocampus of WT and WT + MgT mice (*n* = 6/group) obtained by Western blots following behavioral stimulation. Two tailed unpaired *t*-test. Error bars show SEM. * *p* < 0.05.

We have previously shown that MgT treatment upregulates NR2B-containing NMDARs signaling and increases synapse density in the hippocampus of wild type young and aged rats [18]. The intriguing stabilization of BACE1 expression by MgT prompted us to investigate if this effect of MgT is specific to the Tg mice or MgT might also regulate BACE1 expression in WT mice. We quantified the expression of BACE1 and NR2B (as a positive control) in WT mice treated with MgT (sacrificed at age of 18 month, MgT treatment was given for 12 months). Mice were sacrificed under basal (home-cage) conditions or following environmental enrichment-based stimulation. As expected, MgT treatment upregulated NR2B expression in the hippocampus of WT mice (Figure 6A). However, BACE1 expression was indifferent between WT and WT + MgT mice under basal conditions and following behavioral stimulation (Figure 6B). These results suggest that elevation of brain magnesium stabilizes BACE1 expression only in the Tg mice.

### Reversal of learning and memory deficits and synapse loss in aged APPswe/PS1dE9 transgenic mice by MgT treatment

**Figure 7:**
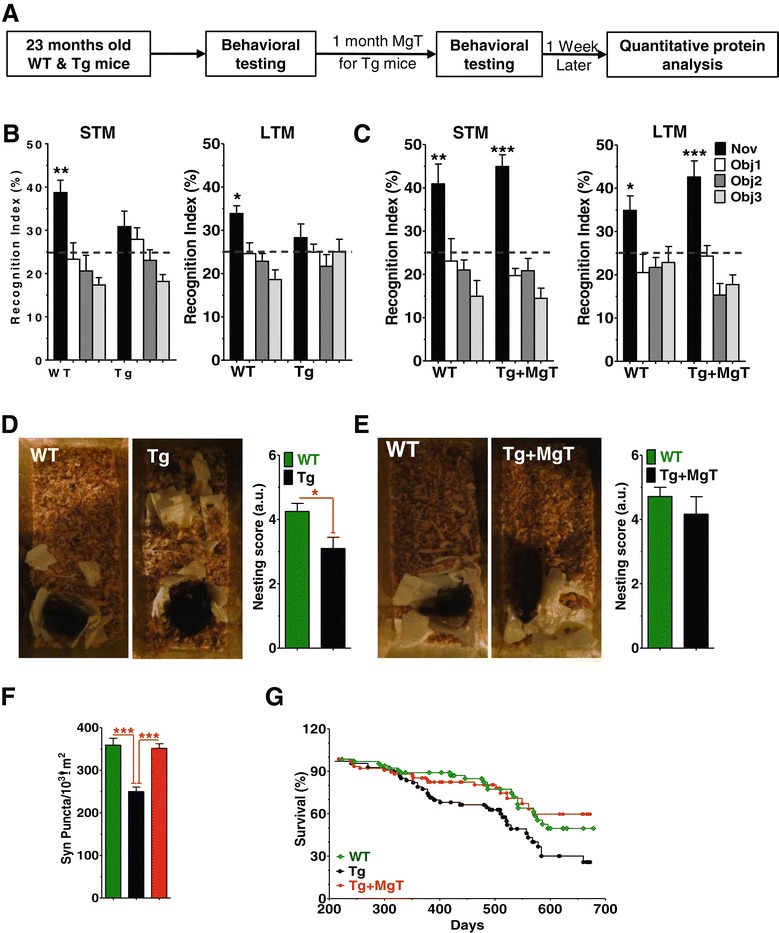
**Reversal by MgT treatment of behavioral and structural deficits in aged APPswe/PS1dE9 mice (Tg mice) and prevention of their premature death. (A)** Experimental design to test whether MgT treatment can reverse behavioral deficits in aged Tg mice. **(B)** Recognition index of the novel object (Nov) and other familiar objects (Obj 1–3) during STM (left) and LTM (right) tests in the NORT task. WT (*n* = 10, ANOVA, STM: *p* < 0.0001; LTM: *p* < 0.0001) and untreated Tg (*n* = 8). **(C)** Same as **(B)** and on the same mice but NORT tests were re-conducted after MgT treatment was given to Tg mice for 1 month. ANOVA revealed significant differences in WT (*n* = 8, STM: *p* < 0.001; LTM: *p* < 0.05) as well as in MgT-treated aged Tg mice (*n* = 7, STM: *p* < 0.0001; LTM: *p* < 0.0001). Dashed lines represent chance levels of performance (25%). ANOVA was followed by Bonferroni’s *post hoc* test. **(D)** Nest construction social behavior in WT (*n* = 10) and untreated Tg mice (*n* = 10). **(E)** Same as **(D)** and on the same WT (*n* = 7) and Tg (*n* = 6) mice, but after MgT treatment was given to Tg for 1 month. **(F)** Density of synaptophysin positive terminals (Syn Puncta). Data from untreated aged Tg mice (from Figure 2B) were inserted (black bars) to show the effects of the 1 month treatment. ANOVAs (Table 2) were followed by Bonferroni’s *post hoc* test. Error bars show SEM. * *p* < 0.05, ** *p* < 0.01, *** *p* < 0.001. **(G)** Survival curves of WT (*n* = 65), Tg (*n* = 68) and Tg + MgT (*n* = 76) over 678 days of lifespan (MgT treatment started at 6 months of age). Log-rank Mantel-Cox test revealed significant difference between Tg mice and WT (*p* < 0.05) as well as Tg + MgT (*p* < 0.01).

Our previous study demonstrates that elevation of brain magnesium can promote synaptogenesis and enhance NMDAR signaling in aging rats [18]. If synapse loss and dysfunction in NMDAR signaling in AD is also reversible, MgT treatment should be effective in reversing memory deficits even when it is given at the end-stage of AD-like pathological progression in Tg mice. To test this possibility, we prepared another group of untreated Tg, tested their memory abilities at 23 month of age, treated them with MgT (1 month) and reevaluated their memory abilities. As expected, before treatment, Tg mice did not show any preference toward the novel object in the STM and LTM tests, in contrast to aged WT (Figure 7A and B). After 1 month of MgT treatment, strikingly, the same Tg mice exhibited significant improvement in their performance on the memory tests (Figure 7A and C). To further prove that MgT treatment is effective in restoring cognitive abilities in end-stage Tg mice, we evaluated the nest construction abilities (a social behavior) in Tg mice before and after MgT treatment in a separate group of mice (23 months old). Before MgT treatment, the Tg mice exhibited significant impairment in nest construction behavior compared with aged WT (*p* < 0.05, Figure 7D). Following only 1 month of MgT treatment, nest construction behavior was restored in Tg mice (*p* = 0.2, Figure 7E). These data demonstrate that learning and memory deficits in the Tg mice might be reversible even at the end-stage of AD-like pathological progression. However, we like to point out a limitation of the above behavioral experiments: the number of animals is relatively low such that the conclusion drawn from these behavioral data might be subjected to potential statistical errors. The lower number of animal in the above experiments was due to poor survival of Tg mice during aging (see below), although we prepared a large number of mice at the beginning of the experiments (see Methods). We took two measures to reduce the potential statistical errors. First, cognitive functions were evaluated by using different behavioral tasks. Second, the memory and nesting behaviors were evaluated in the same groups of Tg mice before and after MgT treatment. Such experimental design should give more confidence in the observed effects of MgT in Tg mice.

To check whether the recovery of memory and social behavior in Tg mice was associated with restoring the synaptic density, we quantified synaptophysin labeled presynaptic terminals in aged WT mice and aged Tg mice with 1 month of MgT treatment (perfused at age of 24 month). Strikingly, in aged Tg mice with 1 month of MgT treatment, the presynaptic terminal density in hippocampal DG was restored to a level comparable to that of aged WT (p = 0.7, Figure 7F, for ANOVA analysis see Table 2). These data suggest that elevation of brain magnesium reversed the behavioral and structural deficits even at the end-stage of the AD-like pathological progression in Tg mice.

Besides the progressive impairment in cognitive abilities, the lifespan of Tg mice was significantly lower than that of WT. MgT treatment prevented the premature death of Tg mice (Figure 7G). This longevity with more normal cognitive function highlights the overall beneficial effects of MgT treatment on body health, at least in mice.

## Discussion

In our previous study, we show that elevation of brain magnesium enhances learning and memory abilities of young and aging rats [18]. Here, we show that elevating brain magnesium was effective in preventing/reversing learning and memory deterioration in APPswe/PS1dE9 transgenic mice, a model of AD-like pathologies. Comparison of synaptic signaling pathways among WT, Tg, and Tg + MgT mice reveals that failure in activating NMDAR signaling is among the major AD-like pathological progression in the APPswe/PS1dE9 mice. The recording studies from hippocampal slices illuminate how elevation of brain magnesium might exert its neuroprotective effects on NMDAR signaling. Unexpectedly, we found that elevation of brain magnesium could prevent dysfunctions in APP metabolism by stabilizing the expression of BACE1 in APPswe/PS1dE9 mice, which might also contribute to the prevention of memory deterioration by MgT treatment. The most striking finding in the current study is that elevation of brain magnesium was effective in restoring synapse density at the end-stage of AD-like pathological progression in APPswe/PS1dE9 mice, which might be responsible to the restoration of cognitive functions.

### How does elevation of brain magnesium protect NMDAR signaling in the APPswe/PS1dE9 transgenic mice?

How dysfunctions of APP metabolism lead to synaptic dysfunctions/loss has been extensively studied [19]. The NMDAR signaling pathway is likely to be one of the primary mechanisms underlying memory deficits in the APPswe/PS1dE9 mice, as NMDARs play a pivotal role in memory processes [27]. A recent study shows that protection of NMDARs is sufficient to ameliorate the memory deficits in an animal model of AD [29]. In the current study, activation of the NMDAR signaling was dramatically impaired in APPswe/PS1dE9 mice. This deficit is likely to be caused by dysfunctions in Ca^2+^ homeostasis in these mice. Cytosolic Ca^2+^ level is hypothesized to be elevated during AD pathologies [30]. Aβ can increase Ca^2+^ entry into the cell either by functioning as a Ca^2+^ channel or by activating plasma membrane Ca^2+^ channels [31,32], which might contribute to elevation of cytosolic Ca^2+^ levels under basal conditions in APPswe/PS1dE9 mice [33]. The increase in intracellular basal Ca^2+^ induces upregulation of calpain/calcineurin/Cdk5 neurodegenerative signaling [34]. These signaling molecules are known as the major downregulators of NMDARs for review see [35]. Hence, the downregulation of NMDARs by elevated Aβ, might be mediated by the upregulation and/or overactivation of calpain and/or calcineurin [28].

In MgT-treated APPswe/PS1dE9 mice the NMDAR signaling pathway was indifferent from WT mice. We hypothesize that Mg^2+^ might inhibit and/or downregulate calcineurin to prevent downregulation of NMDAR signaling (Figure 4F). In our previous studies, we demonstrated that elevation of [Mg^2+^]_o_ reduces [Ca^2+^]_i_, which was associated with NMDARs activation under resting-membrane potentials [17]. This reduction in [Ca^2+^]_i_ triggers homeostatic upregulation of NR2B-containing NMDARs in vitro [17] and in intact animals [18]. In the current study, we show that calcineurin is one of primary molecules mediating the effects of elevating [Mg^2+^]_o_. Since both Aβ and [Mg^2+^]_o_ regulate NMDARs by modulating the activity of calcineurin, we propose that the protective effects of [Mg^2+^]_o_ on NMDARs in high Aβ background might be largely mediated by preventing overactivation/overexpression of calcineurin. In support of the role of overactivation of calcineurin in AD-like pathologies, inhibition of calcineurin alone ameliorates the AD-like pathologies in AD mouse model [36].

### How could elevation of brain magnesium lead to downregulation of BACE1?

Intriguingly, elevation of brain magnesium not only protected NMDAR signaling, but also reduced the expression of BACE1, a critical enzyme that cleaves APP. Recent studies suggest that dysfunctions in APP cleavage by BACE1 might be the primary factor underlying the synapse loss and memory deficits in an AD mouse model [3] and the degeneration of cortical neurons in vitro [4]. Inhibition [3] or partial reduction of expression [37,38] of BACE1 rescues synaptic/memory deficits in mouse models of AD. Therefore, the reductions in BACE1 expression by MgT treatment could contribute to the prevention of synapse loss and improvement of cognitive function in the APPswe/PS1dE9 mice. In the current study, the exact molecular mechanisms underlying prevention of BACE1 overexpression by MgT treatment remain elusive.

Based on the above interpretations, we attributed the positive effects of MgT treatment on cognitive abilities in Tg mice to its protecting actions on the NMDAR signaling pathway and/or stabilizing of BACE1 expression. However, several studies reported increased incidence of epileptic seizures in AD patients [39] and in mouse models of AD [40]. Recently, it was shown that low doses of the antiepileptic drug, levetiracetam, normalize neuronal activity and reverse cognitive deficits in a mouse model of AD [41]. Acute infusions of high doses of Mg^2+^, which elevate blood Mg^2+^ concentration by ~ 2.5 – 4 folds, are effective in reducing seizures acutely in the clinical settings [42,43]. This seizure attenuation is due to direct blockage of presynaptic calcium channels by Mg^2+^ [44,45]. Therefore, an alternative interpretation of the positive effects of MgT treatment is that elevation of brain magnesium increases calcium channels blockage, resulting in reductions in brain seizures. However, for such mechanism to become effective, the Mg^2+^ concentration in blood has to be elevated significantly. In the current study oral administration of MgT only elevates plasma magnesium by ~ 0.1 mM (Figure 1G) and brain magnesium by 15% (Figure 1F). Since the gating of NMDARs is very sensitive to elevations of extracellular Mg^2+^ concentrations [17], the neuroprotective effects of MgT treatment are more likely to be mediated by its action on NMDARs.

### Magnesium concentration and synaptic function

Our studies demonstrate that an increase in magnesium intake enhances memory in young rats, reverses memory decline in aged rats [18] and prevents memory deterioration a mouse model of AD (the current study). However, it is intriguing that following long-term magnesium supplementation, CSF Mg^2+^ concentration only increases by 15% [18] and total magnesium in brain increased by 30% (percentage of Tg mice, Figure 1F). Can small increases in [Mg^2+^]_CSF_ have major impact on synapse density? In a separate study, we found that increase extracellular Mg^2+^ by 15% leads to ~50% increase in synapse density in cultured hippocampal synapses (unpublished observations). These data suggest that hippocampal synapse density might be very sensitive to small changes in extracellular Mg^2+^ concentrations. Under normal physiological conditions, whole body magnesium is tightly regulated by kidney function. Daily fluctuation of plasma magnesium associated with food intake is less than 0.1 mM above a baseline of 0.7 mM [46]. Brain magnesium is supposed to be more stable as the blood–brain barrier isolates the brain from daily fluctuations in blood magnesium. Therefore, despite the high sensitivity of the synapses to Mg^2+^ concentration, synapse density is likely to be stable under physiological conditions. On the other hand, if brain magnesium is reduced under pathological conditions, it might have profound impact on synapse density and memory function. Interestingly, in the hippocampus of AD patients, the total magnesium level is reduced by 18% [20]. Therefore, restoration/elevation of brain magnesium in AD patients might be beneficial for ameliorating the cognitive deficits of AD.

## Methods

### Experimental animals

Six hemizygous transgenic (Tg) male mice (APPswe/PSEN1dE9)-85Dbo (referred to as ‘Tg mice’) and six non-carrier female mice were obtained and bred in Peking University’s laboratory animal center according to protocol provided by the supplier (The Jackson Laboratories). In the breeding colony, we generated 1000 mice in total, which were genotyped routinely to identify the Tg mice. Overall, ~ 250 male wild type (WT) and ~ 250 male Tg mice we prepared and majority of them were used in the current study. Animals were housed individually in a controlled environment (temperature 21 ± 1°C, humidity 50 ± 10%) under inverted light cycle (light off 9:00 AM to 9:00 PM). Behavioral experiments were performed under red dim light. All experiments were performed on male mice except Western blot experiments in which female mice were used (WT: n = 48; Tg: n = 48). To conduct molecular, cellular and structural analysis, animals were sacrificed at ages of 15, 18, 23 or 24 months old (for details on each experiment see Results section). Experiments involving animals were approved by Tsinghua and Peking Universities Committees on Animal Care.

### Treatment with Magnesium-L-Threonate

Magnesium-L-Threonate (MgT, Magceutics, Inc., CA, USA) treated Tg and/or WT mice received the MgT treatment via drinking water at a dose of ~910 mg/kg/day (~75 mg/kg/day elemental magnesium). This dose was determined by scaling the minimum effective dose in rats, which we described before [18]. To monitor the dose of MgT (mg/kg/day), water intake and body weight were measured on daily basis (8:30 pm). The amount of MgT necessary to reach the target dose was calculated, based on the body weight, and dissolved in the daily drinking water (~6 ml/day/mouse) of individual animals. The MgT treatment was started when the mice were at the age of 6 months and lasted until animals were sacrificed, excluding the end-stage treatment experiments (Figure 7A-F) during which MgT treatment was given to Tg mice at age of 23 months for 1 month only. Untreated WT and Tg mice received tap water. All mice were maintained on standard food containing 0.15% elemental magnesium.

### Magnesium measurement in body tissues and fluids

To determine the total magnesium content (ionized and non-ionized) in different body tissues, mice were transcardially perfused and then different body tissues; such as kidney, liver, muscles, fur, heart, and brain, were dissected. Total magnesium content in different tissues was measured using inductively coupled plasma optical emission spectroscopy (ICP-OES, Chemical Analysis Center, Tsinghua University). To determine the free Mg^2+^ concentration in the plasma and intracellular compartment of red blood cells, blood samples were collected from the orbital sinus, centrifuged and plasma was collected for measurement immediately. Mg^2+^ concentration in the plasma was measured using Calmagite chromometry as we described before [18]. The intracellular Mg^2+^ concentration in red blood cell (RBC, Figure 1H) was determined by flow cytometry (Center for Biomedical Analysis, Tsinghua University). The fluorescent optical density (OD) was used as an indicator of the Mg^2+^ concentration in RBC. The presented data represent the average of OD of 10000 RBCs for each mouse.

### Morris water maze (MWM)

Spatial reference memory was assessed using a modified version of the Morris water maze [47]. The pool was a circular metal tank, 100 cm in diameter, 50 cm deep, filled to a height of 35 cm with water. Water temperature was maintained at 21 ± 1°C degree. An acrylic platform (10 cm in diameter) was placed inside the pool, its upper surface 1 cm below the surface of the water, so that a mouse inside the pool would be unable to locate it visually. The pool was set in a moderately lit, circular enclosure made with black curtains. Four cues with different shape and color were placed within the maze, and other four different cues were placed externally on the curtains. These cues remained unchanged throughout the testing period. The combination of proximal and distal cues was used based on pilot experiments during which we tested the sensitivity of WT mice to distal and proximal cues. Under our experimental conditions the optimal learning curve for naïve WT mice (declining from ~ 60 s to ~ < 20 s over the time course of training) was obtained upon combining proximal and distal cues. Two protocols were used: First, at 7 months of age, mice received the visible platform training session for one day (day 0). This session was followed by hidden platform training session for five days with five trials per day (60 s per trial, 1 h inter-trial interval). Each mouse was placed into the water by hand, so that it faced the wall of the pool, at one of four starting positions. The sequence of these positions was randomly selected. The platform was set in the middle of one quadrant, equidistant from the center and the edge of the pool. If the mouse found the platform, it was allowed to remain there for 30 s and was then returned to its home cage. If the mouse was unable to find the platform within 60 s, it was guided to and placed on the platform for 30 s, the trial was terminated, and the maximum score of 60 s was given. In each trial, the latency to locate the hidden platform was recorded using Noldus video tracking system (Ethovision). The test trial (the memory retention test) was carried out 24 h after the last trial of the training session. During the test trial, the platform was removed and each mouse was put into the pool for 60 s. The total time spent in the target quadrant (where the platform had been located during the training trials) was measured using the same video system. Experiments and video analysis were performed by experimenters who were not aware of the genotype and the treatment of each mouse.

Second, at 15 months of age, we modified the water maze protocol to increase the task’s difficulty. The tank-diameter was increased to 120 cm with 12 cm diameter platform under open-environment (curtains were removed) around the water maze. Mice were trained for 4 days (6 trials per day, 60 s per trial, 1 h inter-trial interval).

### Novel-object recognition task

A modified version of the novel-object recognition test [48] was used to evaluate short-/long-term recognition memories. The apparatus consisted of a square arena (50 × 50 × 20 cm) made of polyvinyl chloride with white walls and floor. An overhead camera and a video recorder were used to monitor and record the animal’s behavior for subsequent analysis. Two days before the experiment, all mice received two sessions of habituation to the arena and test room 10 m per session/day. At day 3, each mouse was first placed in the center of the box, and exposed to four identical objects for 5 m (sample phase). Then the mouse was returned to its cage. For short-term memory, a 5 m retention interval was used, during which one of the objects was replaced by a new novel object. The objects were placed in the same locations as the previous ones. The mice were placed back in the box and exposed to the three familiar objects and to the novel object for further 5 m (test phase). During this phase, the mouse explored each of the four objects. The exploration of an object was defined as directing the nose to the object at a distance of 2 cm and/or touching it with the nose and forepaws. Turning around the object without direct exploration was not considered. The session was recorded on video, and the frequency of object exploration was subsequently measured by the experimenters who were blind to the treatment and genotype of each group. Recognition index was calculated as percentage ratio of time of each object over total exploration time. The box and objects were cleaned with 20% ethanol between trials to prevent the build-up of olfactory cues. For long-term memory same procedure was used but the retention interval was 24 h and completely different objects were used.

### Nest construction test

Mice were individually housed in plastic cages with approximately 1 cm of woodchip bedding lining the floor. Two hours after the onset of the dark phase, individual cages were supplied with a 20 cm × 20 cm piece of paper towel torn into approximately 5 cm × 5 cm squared pieces. Cages were observed for the next 12 h. Pictures were taken for documentation before the evaluation. Nest construction was scored along a system as followed: 1 = no biting or tears on the paper, 2 = mild biting and/or tears without gathering, 3 = moderate biting and/or tears on the paper with moderate gathering of pieces, 4 = the majority of papers torn and gathered in a good manner, and 5 = the vast majority of paper torn into approximately 1 cm pieces and grouped into a corner of the cage. Twenty-four hours after the first nest construction test, MgT treatment of the Tg mice started and lasted for 1 month, whereupon the second test was conducted. Mice were tested in counterbalanced groups of mixed genotypes. Nest construction was scored by experimenters who were unaware of the treatment and genotype of each group.

### Brains preparation for electron microscopy and staining of presynaptic puncta and amyloid plaques

Mice (under home cage conditions) were anesthetized with chloral hydrate and perfused transcardially with physiological saline followed by 4% paraformaldehyde in 0.1 M phosphate buffer (pH 7.4). The left hemispheres were immersed in the same fixative overnight, embedded in paraffin, and were prepared for presynaptic puncta and amyloid plaques immunostaining. The right hemispheres were prepared for transmission electron microscopy.

### Transmission electron microscopic analysis

The right hemisphere was sectioned (500 μm thick coronal) to expose hippocampus. After brief rinse in 0.1 M sodium cacodylate buffer (SCB, pH 7.4), samples were immersion-fixed overnight in 2.5% glutaraldehyde and 2% paraformaldehyde in 0.1 M SCB containing 1% tannic acid. After rinsing in SCB and postfixation for 1 h in 1% osmium tetroxide, 0.1 M SCB, the hippocampal sections were dehydrated using a series of acetone dilutions and were flat-embedded in an Epon resin. Semithin sections (500 nm) stained with toluidine blue were used to locate and trim granule cell layer and molecular layer of dentate gyrus in the hippocampus. Ultrathin sections (60 nm) stained with uranyl acetate and lead citrate, were examined with a JEOL-1200EX transmission electron microscope (Japan Electric Optical Laboratory) operated at 80 kV. Two sets of images were randomly captured at magnifications of 12,000 and 30,000, respectively. Scale bars were taken from scans of original electron micrograph negatives. Quantitative analysis was conducted on the digital EM micrographs of the same magnification from the outer molecular layer of the hippocampus dentate gyrus from all mice. The measurement was performed by experimenters who were not aware of the genotyping/treatment and was assisted by MetaMorph software (Molecular Devices). Synapses were identified on the micrographs by the presence of postsynaptic density (PSD) and at least two synaptic vesicles in the axon terminal in close proximity. For the analysis of the synaptic density, the sets of lower magnification micrographs were used (24–26 micrographs for each mouse).

### Estimation of density of presynaptic puncta

Density of synaptophysin- /glutamic acid decarboxylase 65kD (GAD65)-immunoreactive presynaptic terminals were quantified as we previously described for synaptophysin positive puncta [18]. Coronal sections (6 μm thick) from the left hemisphere were deparaffinized in xylene and rehydrated using a descending ethanol series followed by epitope retrieval with citrate buffer. After blocking with 5% normal serum (from the host of the secondary antibody) in PBS with 0.1% Triton X-100, tissue sections were incubated overnight with the primary antibody (see Table 3 for detailed information on each antibody) in PBS with 3% serum at 4°C. After washing in PBS, sections were incubated with CF^488^-conjugated (Biotium Inc., CA, USA) secondary antibody (Table 3). After washing with PBS, sections were coverslipped with anti-fade mounting medium (Vectashield). Slides were coded until the completion of data analysis. Stained brain sections were imaged with Olympus IX-70 confocal microscope with the 60 ×; water lens (N/A = 1.2) at zoom × 3, generating an image with 78.6 × 78.6 μm dimension. Serial z-sectioning was performed (thickness of 0.6 μm) and the best three z-sections (with highest number of puncta) were collected and merged into a single image. Therefore, volume of brain tissue per image is ~78.6 × 78.6 × 1.8 μm^3^. The number of synapses in the DG and CA1 subregions was estimated from the obtained images using Image-Pro-Plus software (Media Cybernetics). Background levels were equalized and special filters to separate fluorescent puncta were applied. Settings, for each image, were adjusted to maximize the number of detected fluorescent puncta. Mean puncta number per 1000 μm^2^ was used as an estimate of the presynaptic puncta density.

**Table 3:** **Primary and secondary antibodies**

**Experiments**	**Primary antibodies**	**Host**	**Dilution**	**Source**
Quantitative immunostaining	Synaptophysin	Mouse	1:500	Millipore
	GAD65	Rabbit	1:500	Millipore
Western blot	NR2B	Rabbit	1:6000	Santa Cruz
	pCamkII (THr286)	Rabbit	1:1000	Cell Signaling
	CamkII	Rabbit	1:1000	Cell Signaling
	pCreb (Ser133)	Rabbit	1:50	Cell Signaling
	Creb	Rabbit	1:1000	Cell Signaling
	GAPDH	Rabbit	1:6000	Cell Signaling
	BACE1	Rabbit	1:500	Cell Signaling
	6E10 (β-CTF)	mouse	1:6000	Signet
	hsAPPβ	Rabbit	1:50	IBL
**Experiments**	**Secondary antibodies**		**Dilution**	**Source**
Quantitative immunostaining	CF™488 Conjugated Goat anti-Mouse IgG(H + L)		1:200	Biotium
	CF™488 Conjugated Goat anti-Rabbit IgG(H + L)		1:200	Biotium
Western blot	HRP-conjugated Goat anti-Rabbit IgG(H + L)		1:20000	ZSGB-BIO
	HRP-conjugated Goat anti-Mouse IgG(H + L)		1:25000	CST

### Amyloid plaque immunostaining

Plaque burden was assessed by immunohistochemistry on paraffin-embedded sections using monoclonal anti-Aβ antibodies 4G8 and/or 6E10 (Signet), as described previously [22]. Prior to immunostaining, sections were deparaffinized in xylene and washed in 100% ethanol, 95% ethanol, 70% ethanol, and water. Endogenous peroxidase activities in sections were quenched by 10 m incubations in 3% H_2_O_2_ in methanol followed by epitope retrieval with citrate buffer and 5 m incubation in 85% formic acid. After rinse in PBS, nonspecific epitopes were blocked with normal horse serum. Then sections were incubated with primary antibody 4G8 and/or 6E10 (1:500 dilution) in PBS with 3% BSA and incubated over night at 4°C in a humid chamber. Sections were then washed in PBS and incubated with biotinylated secondary antibodies and avidin-biotin-peroxidase as described by the manufacturer (Vectastain Elite ABC Kit) and visualized with diaminobenzidine tetrachloride (DAB). Stained sections containing hippocampus were examined with a Leica DM IRB inverted research microscope, using 5 × objective, and digital images were captured with a Retiga 2000R digital camera (Qcaputure). The areas covered by amyloid plaque in the hippocampal formation and frontal cortex were calculated using Image-Pro Plus. The area of interest was manually outlined, and the percentage of area occupied by Aβ plaque, in the hippocampus and frontal cortex, was calculated.

### Aβ42 and Aβ40 concentrations in the cerebrospinal fluid

To determine the concentration of Aβ42 and 40 monomers in the cerebrospinal fluid, mice were anesthetized with chloral hydrate (400 mg/kg, i.p.) and then the CSF was manually obtained from the cisterna magna by the interruption of the atlanto-occipital membrane using a recording pipettes (diameter 0.5 μm). CSF samples (8 μl/mouse) were collected and stored at −20°C until measurement was performed. The Aβ42 and Aβ40 monomers concentrations were determined using ELISA kit (human β amyloid [1–40] and [1–42] kits, Wako, Osaka, Japan). The procedure was performed with complete adherence to the manufacturer’s instructions.

### Environmental enrichment-based stimulation for Western blot analyses

For quantitative Western blot experiments (Figures 3, 5C-E and 6), mice were exposed to environmental enrichment-based behavioral stimulation for 24 h before being sacrificed. Mice were housed individually in standard cages (300 cm^2^ floor space), the stimulus of enrichment was divided into two phases: grouping (for 21 h) and physical-training (for 3 h). During the grouping phase, mice were group-housed in a large cage (5–6 mice/cage, 70 cm*60 cm*30 cm) without any running wheels or toys. During the physical-training phase the mice in the large cage were given running wheels, play tubes, and wood boxes.

### Western blot

Frozen (−80°C) hippocampal tissues were homogenized and equal amounts of proteins were resolved on polyacrylamide gel, then transferred to PVDF membranes (Millipore). Membranes were blocked and then probed with primary antibodies against NR2B (H-50, sc-9057, from Santa Cruz Biotechnology), BACE1 (D10E5), CREB, pCREB, CaMKII, pCaMKII, GAPDH (all from Cell Signaling Technology), sAPPβ (IBL) or β-CTF (6E10, Signet, see Table 3) overnight at 4°C. Membranes were then incubated with an HRP-conjugated secondary antibody (ZSGB-BIO or CST, see Table 3) at room temperature. Protein bands were detected by ECL detection reagent (RPN2232; GE Healthcare) and captured on an autoradiography film (Kodak). Integrated optical density was determined using Image-Pro Plus software 6.0 (Media Cybernetics). Standard curves were constructed to establish that we operated within the linear range of the detection method. Co-detection of GAPDH on the same membrane served as a loading control. Quantitative analysis was performed by experimenters blind to the treated and/or genotype group.

### *In vivo* and *in vitro* recording

#### Input–output relationship in CA1 synapses in vivo

Mice were anesthetized with urethane (2 g/kg, i.p.). The animals were fixed in a stereotaxic apparatus (Narishige SN-2, Japan). Field postsynaptic potentials (fPSPs) were recorded from the stratum radiatum in CA1 following electrical stimulation of Schaffer collateral–commissural pathway. The optimal electrode placement was determined using electrophysiological criteria as described before [49]. The recording electrode was positioned at 2.3 mm posterior to bregma, 1.75 mm lateral to midline and the depth of recording electrode was about 1.6 mm from dura. The stimulating electrode was positioned 1.7 mm posterior to bregma and 1.6 mm lateral to midline and about 1.8 mm from dura. The positions of the recoding and stimulation electrodes were confirmed by trailing the electrodes in brain sections from representative animals. A single square pulse of voltage at low frequency (0.066 Hz, 0.2 ms duration) was used to evoke fPSPs and the intensity of the test stimulus was adjusted to produce ~50-55% of maximum response.

#### Slice preparation for in vitro recordings

Acute coronal slices of hippocampus (400 μm thick) were prepared from 4 weeks old male C57BL/6 mice as described before [50]. Briefly, slices were transferred and submerged in a recovery chamber containing oxygenated (95% O_2_ and 5% CO_2_) artificial cerebrospinal fluid (ACSF, mM): 125 NaCl, 2.5 KCl, 2 CaCl_2_, 1.2 or 0.8 MgCl_2_, 26 NaHCO_3_, 1.25 NaH_2_PO_4_, 25 glucose. The extracellular magnesium concentration ([Mg^2+^]_o_) varied according to the experimental conditions. Slices were incubated in ACSF containing either physiological extracellular magnesium concentration ([Mg^2+^]_o_ = 0.8 mM) or high [Mg^2+^]_o_ (1.2 mM), at 32°C for 1 h and then at room temperature for 3 h before recording. Irrespective of the pre-incubation conditions, the recording was performed under 1.2 mM [Mg^2+^]_o_ to avoid any potential acute effect of elevating [Mg^2+^]_o_.

#### Amyloid-β and FK506

Application of Amyloid-β and FK506 was done as described before [28]. Briefly, Amyloid-β42 monomer (1 μM, Amyloid β-Protein Fragment 1–42, sigma) and/or FK506 (10 μM, Sigma) were applied into the ACSF after the 3 h recovery period and for 1 h before recording started.

#### Whole-cell recordings in vitro

Experiments were performed in a recording chamber on the stage of a microscope (Eclipse FN1, Nikon) with infrared DIC optics (IR1000, DAGE-MTI) for visualizing whole-cell patch-clamp recordings. EPSCs were recorded from CA1 pyramidal neurons, using an EPC-9 amplifier with Pulse v8.65 software (HEKA Elek., Lambrecht, Germany), while stimulating the Schaffer collateral-commissural pathway. A moderate constant amplitude stimulus, by Stimulus Isolator A365 (World Precision Instruments, Sarasota, FL USA) with bipolar tungsten stimulating electrode, was used to stimulate axonal fibers and a population of synapses. The extracellular solution also contained picrotoxin (100 μM, Sigma) to block fast GABAergic inhibition. The recording pipettes (3–5 MΩ) were filled with solution containing (mM): 130 Cs-gluconate, 4 NaCl, 0.5 MgCl_2_, 5 EGTA, 10 HEPES, 5 MgATP, 0.5 Na_3_GTP and 5 QX-314 (adjusted to pH 7.3 with CsOH and osm. 290–295 with double distilled water).

For EPSC_NMDA_/EPSC_AMPA_ experiments, after whole-cell patch-clamp, neurons were voltage clamped at −70 mV to record the EPSC_AMPA_, and then were clamped at +50 mV to record the EPSC_NMDA_ (with liquid junction potential correction). EPSCs were induced by repetitive stimulations at 0.1 Hz. The peak amplitude of EPSC_AMPA_ was determined at the peak of the EPSCs recorded at −70 mV, and the peak amplitude of EPSC_NMDA_ was determined at +50 mV and 200 ms after stimulation. The I_NMDA/AMPA_ ratio was calculated from the recorded data of individual neurons.

### Statistics

Learning curves in the water maze task were analyzed using Two-way ANOVA (treatment x trials) repeated measure. The water maze memory tests, NORT, magnesium contents in brain tissue, electron microscopy, fluorescent immunostaining and Western blot data were analyzed using One-way ANOVA. ANOVAs were followed by Bonferroni’s *post hoc* test. All data involved comparing two groups were analyzed using two-tailed unpaired *t*-test. *P* value less than 0.05 was considered statistically significant.

## Additional files

Additional file 1: Figure S1. The raw data for Western Blot in Figure 5C-E. Additional file 2: Figure S2. The raw data for Western Blot in Figure 6.

## Abbreviations

MgT: Magnesium-L-Threonate
AD: Alzheimer’s disease
NMDA: N-methyl-D-aspartate
CREB: Cyclic AMP-Responsive Element-binding protein
BACE1: Beta-secretes 1
sAPP: Soluble beta Amyloid Precursor Protein
β-CTF: Beta- C-terminal fragment
NMDARs: N-methyl-D-aspartate receptors
APP: Amyloid Precursor Protein
Aβ: Amyloid beta
NAP: Neutrophil alkaline phosphatase
NGF: Nerve Growth Factor
BDNF: Brain-derived neurotropic factor
Mg^2+^: Magnesium Ion
Tg: Transgenic
WT: Wild type
ICP-OES: Inductively coupled plasma optical emission spectroscopy
RBC: Red blood cell
OD: Optical density
MWM: Morris water maze
SCB: Sodium CacodylateBuffer
PSD: Postsynaptic density
GAD65: Glutamic acid decarboxylase 65 kD
PBS: Phosphate buffered saline
BSA: Bovine serum albumin
DAB: Diaminobenzidine Tetrachloride
Aβ42: Amyloid-beta 42
Aβ40: Amyloid-beta 40
ELISA: Enzyme-linked immunosorbent assay
PVDF: Polyvinylidene Difluoride
NR2B: N-methyl D-aspartate receptor subtype 2B
pCREB: Phophorylated cAMP-responsive element bindingprotein
CaMK II: Calmodulin-dependent protein kinase II
GAPDH: Glyceraldehyde-3-phosphate dehydrogenase
fPSPs: Field postsynaptic potentials
ACSF: fuid
EGTA: Ethylene glycol tetraacetic acid
HEPES: Ethanesulfonic acid
EPSC: Excitatory Postsynaptic Current
AMPA: α-amino-3-hydroxy-5-methyl-4-isoxazole-propionic acid receptor
ANOVA: Analysis of variance
NORT: Novel object recognition test
DG: Dentate gyrus
GABA: Gamma amino butyric acid
fPSPs: Field postsynaptic potentials
CSF: Cerebrospinal fluid
PS1: Presenilin-1
STM: Short term memory
LTM: Long term memory
CNS: Central nervous system
SEM: Standard error of the mean
RI: Recognition index
IPP: Image pro plus
PSD: Postsynaptic density
OML: Outer molecular layer
NGF: Nerve growth factor
LTP: Long-term potentiation
