# Supplementary material for: Elevation of brain magnesium prevents synaptic loss and reverses cognitive deficits in Alzheimer’s disease mouse model
Source: Mol Brain. 2014 Sep 13;7:65. doi: 10.1186/s13041-014-0065-y (PMC4172865; doi:10.1186/s13041-014-0065-y)
Supplement: Additional file 1: Figure S1. — The raw data for Western Blot in Figure 5C-E. [file 13041_2014_65_MOESM1_ESM.ppt]

## Slide 1
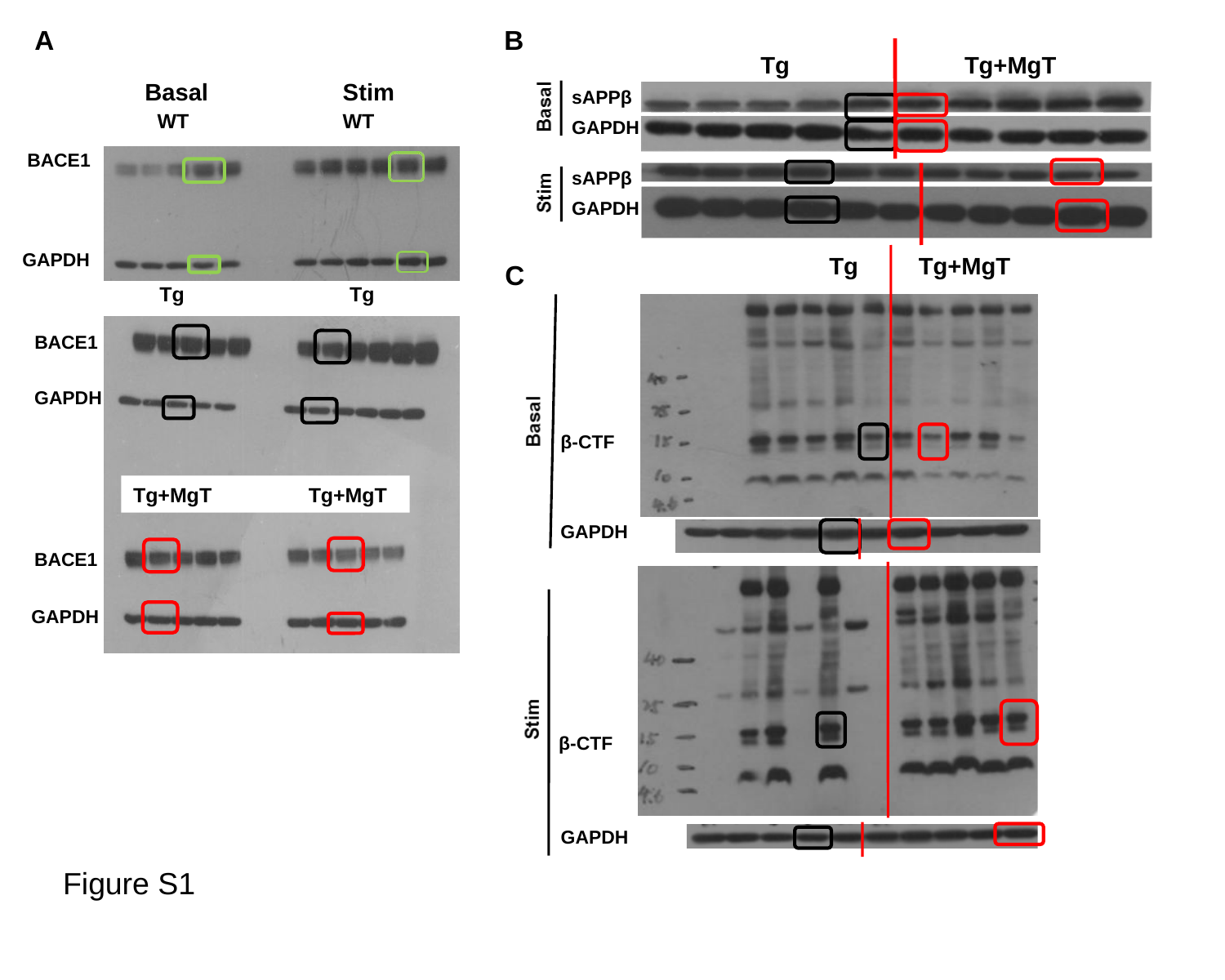

A
B
Tg Tg+MgT
sAPPβ
GAPDH
sAPPβ
GAPDH
Basal Stim
 WT WT
BACE1
GAPDH
 Tg Tg
BACE1
GAPDH
Tg+MgT Tg+MgT
BACE1
GAPDH
 Tg Tg+MgT
C
β-CTF
GAPDH
β-CTF
GAPDH
Figure S1
