# Supplementary material for: Elevation of brain magnesium prevents synaptic loss and reverses cognitive deficits in Alzheimer’s disease mouse model
Source: Mol Brain. 2014 Sep 13;7:65. doi: 10.1186/s13041-014-0065-y (PMC4172865; doi:10.1186/s13041-014-0065-y)
Supplement: Additional file 2: Figure S2. — The raw data for Western Blot in Figure 6. [file 13041_2014_65_MOESM2_ESM.ppt]

## Slide 1
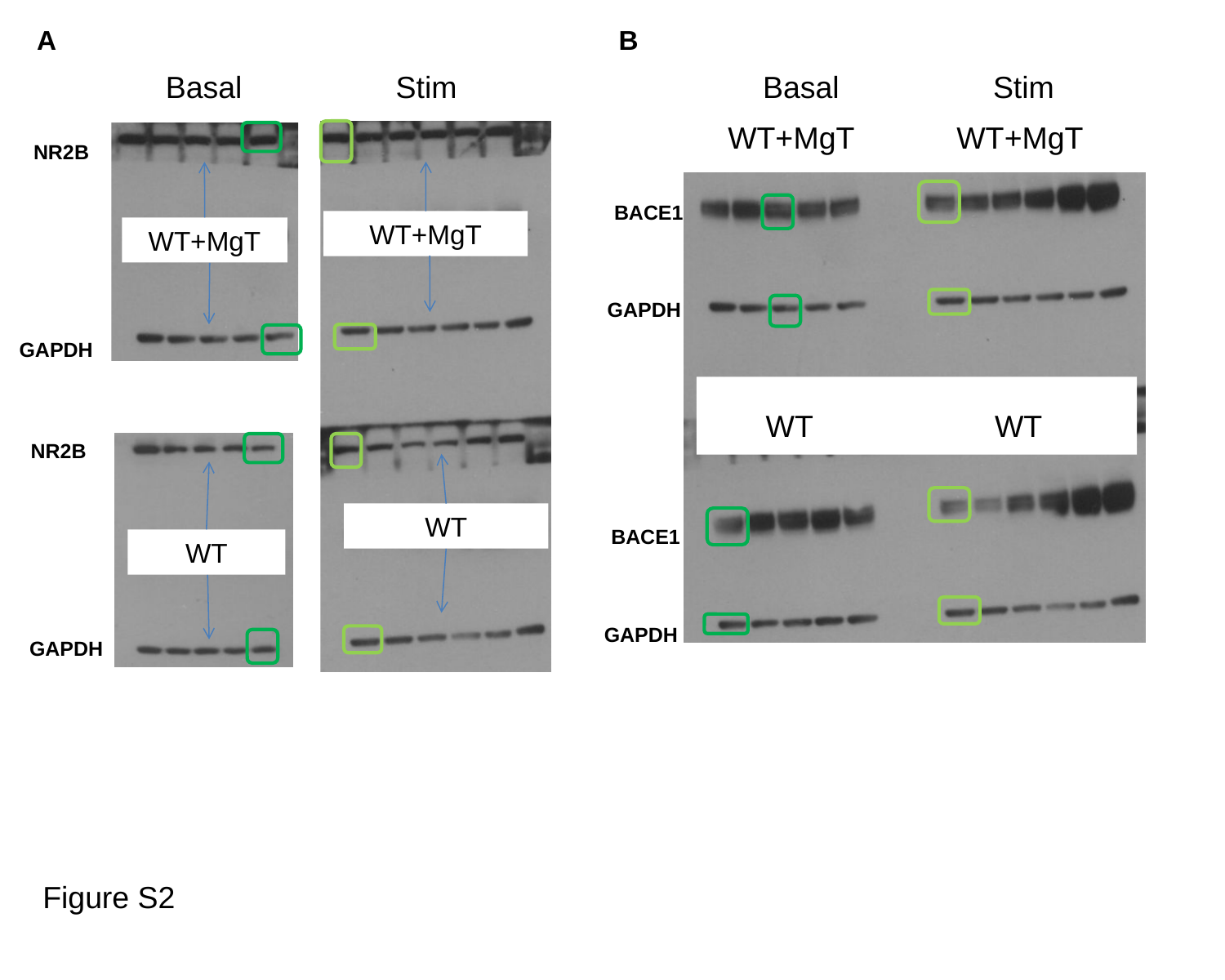

A
B
 Basal Stim
WT+MgT
NR2B
WT+MgT
WT
GAPDH
NR2B
GAPDH
WT
 Basal Stim
WT+MgT WT+MgT
 WT WT
BACE1
GAPDH
BACE1
GAPDH
Figure S2
